# Surveying silicon nitride nanopores for glycomics and heparin quality assurance

**DOI:** 10.1038/s41467-018-05751-y

**Published:** 2018-08-16

**Authors:** Buddini Iroshika Karawdeniya, Y. M. Nuwan D. Y. Bandara, Jonathan W. Nichols, Robert B. Chevalier, Jason R. Dwyer

**Affiliations:** Department of Chemistry, University of Rhode Island, 140 Flagg Road, Kingston, 02881 USA

## Abstract

Polysaccharides have key biological functions and can be harnessed for therapeutic roles, such as the anticoagulant heparin. Their complexity—e.g., >100 monosaccharides with variety in linkage and branching structure—significantly complicates analysis compared to other biopolymers such as DNA and proteins. More, and improved, analysis tools have been called for, and here we demonstrate that solid-state silicon nitride nanopore sensors and tuned sensing conditions can be used to reliably detect native polysaccharides and enzymatic digestion products, differentiate between different polysaccharides in straightforward assays, provide new experimental insights into nanopore electrokinetics, and uncover polysaccharide properties. We show that nanopore sensing allows us to easily differentiate between a clinical heparin sample and one spiked with the contaminant that caused deaths in 2008 when its presence went undetected by conventional assays. The work reported here lays a foundation to further explore polysaccharide characterization and develop assays using thin-film solid-state nanopore sensors.

## Abstract (web summary)

The complexity of polysaccharides significantly complicates their analysis in comparison to other biopolymers. Here, the authors demonstrate that solid-state silicon nitride nanopore sensors can be used to reliably detect native polysaccharides and to perform a simple quality assurance assay on a polysaccharide therapeutic, heparin.

## Introduction

**Fig. 1:**
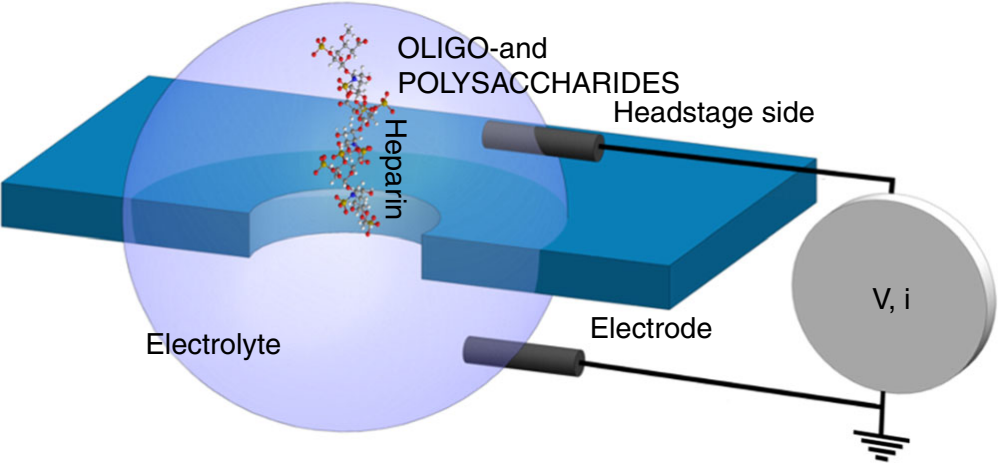
Schematic of the nanopore setup. Voltage-driven passage of a molecule into, across, or through an electrolyte-filled nanopore can be used for analyte detection and characterization

Oligo- and polysaccharides are ubiquitous in nature, with a broad spectrum of roles that includes energy-storage and provision (including as a foodstuff), structural building block (e.g., cellulose), therapeutic function (e.g., the anticoagulant heparin), and a vital part in biological recognition processes^1–11^. Conventional chemical analysis tools are frequently challenged by the daunting complexity of polysaccharide analysis:^12,13^ identification of monomer composition (~120 naturally occurring monomers!) and sequence, monomer linkage types, stereochemistry, polymer length, and degree of polymer branching^13^. These challenges were tragically driven home in 2008 when undetected contamination of the common anticoagulant heparin by a structurally similar adulterant, oversulfated chondroitin sulfate (OSCS), resulted in profoundly adverse clinical consequences in the United States, including ~100 deaths^14–19^. Glycan samples can be challenged by heterogeneity and low abundance in addition to chemical and structural diversity, so while new analysis tools have been broadly called for^12,13,20^, single-molecule-sensitive methods are a particularly compelling goal for glycomics—more so given the absence of sample amplification techniques analogous to PCR for DNA sequencing^21^. Nanopore single-molecule methods have emerged as a powerful tool for characterizing DNA and proteins, including aspects of sequence, structure, and interactions^22–28^. Monomer-resolved length determinations of more prosaic polyethylene glycol samples further buttress the potential of suitably configured nanopore assays for the analysis of polymers with biological utility^29^. The simplest implementation for nanopore measurements places the nanopore—a <100 nm-long nanofluidic channel through an insulating membrane—between two electrolyte solutions (Fig. 1). Ion passage through the nanopore in response to a voltage applied across the pore gives the baseline open pore current, *i*_0_; passage of a molecule into, across, or through the nanopore disrupts this ion flow to give a blocked-pore current, *i*_b_. A discernible current perturbation reveals the presence of an analyte, and the sign, magnitude, and temporal structure of *i*_b_ depend strongly on size and shape of the analyte—and of the nanopore—and on the applied voltage and bulk and interfacial charge distributions. It thus provides insight into analyte presence, identity, and properties, including interactions between the analyte and pore interior or surface^29–32^. Analysis of the resistive-pulse characteristics of a sample offers the potential to glean molecular-level insights, but the *i*_b_ characteristics can also be used more simply as benchmarks in quality assurance assays where atypical *i*_b_ values signal sample impurities.

Much groundwork must be laid, including proof-of-principle experiments, if nanopore methods are to emerge as a tool for glycan profiling—and by extension as a tool for –omics writ-large (spanning genomics, proteomics, and glycomics). Sugar-pore binding, polysaccharides, and enzyme-digested oligosaccharides have been detected using a range of different nanopore platforms from protein to solid-state^33–43^. While solid-state nanopores in thin (~10 nm) membranes have been often portrayed as the preeminent nanopore platform, their use to profile classes of molecules beyond DNA and proteins is in its infancy. These nanopores can be size-tuned^44^ to match analyte dimensions (especially relevant for branched polysaccharides), and when fabricated from conventional nanofabrication materials such as silicon nitride (SiN_x_)^45,46^, offer resistance to chemical and mechanical insult alongside low barriers to large-scale manufacturing and device integration. The potential for integration of additional instrumentation components, such as control and readout electrodes, around the thin-film SiN_x_ nanopore core, is especially compelling^28,45,46^. Recent (nanopore-free) work on recognition electron tunneling measurements on polysaccharides, for example, has reaffirmed the importance of a nanopore development path that values augmented nanopore sensing capabilities^47^. A key question concerning the use of SiN_x_ nanopores for polysaccharide sensing is whether this fabrication material is compatible with sensing glycans, which can exhibit a wide range of chemical and physical properties. The often challenging surface chemistry of SiN_x_ (giving rise to a complex surface charge distribution)^45,46,48^ may lead to analyte-pore interactions that hinder or prevent its use. Variability in polysaccharide electrokinetic mobility arising from differences in molecular structures may exacerbate the effect of these interactions. These issues become particularly important when analyte translocation through a constricted pore is required, such as in transverse electron tunneling measurements^28,47^.

Naturally occurring sodium alginate, with uses in biomedical and food industries, presents an overall negative, but unexceptional, formal charge in neutral pH aqueous solutions. Sourcing variability for alginates that are extracted from seaweed can be as prosaic as molecular weight to more enticing changes in the relative abundances of alginate’s constituent mannuronate (M) and guluronate (G) residues^49^. At a compositional extreme, heparin, the prevalent anticoagulant drug, is the most highly negative charge-dense biological molecule known^50^. This exceptional charge density couples with the demonstrated difficulty, by other methods, of detecting the negatively charged OSCS (molecular weight ~17 kDa^51^) contaminant in a heparin sample^14–17^ to make the analysis of heparin (~16 kDa) and OSCS by nanopore a compelling experimental test with clinical relevance.

The aims of the present work are: (1) to explore using SiN_x_ nanopores for sensing polysaccharides with a range of possibly challenging chemical and physical properties; and (2) to gauge the prospects of a clinically relevant assay to detect the toxic OSCS impurity in heparin. We profile alginates with different properties—**A1** (*M*_*n*_~74 kDa) and **A2** (*M*_*n*_~18 kDa)—to diversify the analyte scope, and change the solution pH to explore the effect of SiN_x_ nanopore surface charge on the electrokinetics of polysaccharide detection. Highly charge-dense heparin further expands the molecular pool, and a change of electrolyte concentration is used to improve the signal levels. Under these solution conditions, we perform analytical determinations of heparin at clinically relevant concentrations and detect OSCS impurities in the heparin sample. Using a simple statistical thresholding algorithm, we detect this impurity using nanopores differing in apparent diameter by as much as 50%.

## Results

### Exploring nanopore polysaccharide sensing using alginates

**Fig. 2:**
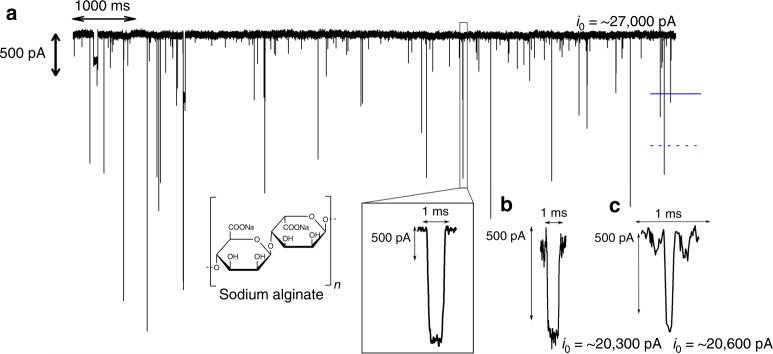
Representative nanopore current trace and events from sodium alginate samples from two different sources. **a** A representative segment of an **A1**-induced current trace using a ~21 nm-diameter pore; the solid blue line marks the most frequent event level, *i*_b_, and the blue dashed line is its mean across all events. The magnified current event is from the same trace. **b** **A2**- and **c** enzyme-digested-**A2**-associated single events through a ~22 nm-diameter pore. All currents were measured in response to a −200 mV voltage difference

Introduction of anionic alginate **A1** (*M*_*n*_~74 kDa; alginate masses determined by viscosity, see Supplementary Methods) into the headstage sample well failed to generate detectable transient current changes when a positive voltage difference (the polarity consistent with purely electrophoretic motion for an anionic analyte) was applied with the analyte in the same well (Fig. 1). Application of a negative potential difference, instead, generated transient current changes (here denoted events) that could be readily differentiated from the open current noise with ~60:1 event-to-noise frequency compared to analyte-free scans. Figure 2 shows a representative time trace of **A1**-induced events, with a characteristic event magnified. The frequency of discrete current blockages associated with the addition of **A1** showed a linear increase with analyte concentration (Supplementary Fig. 1), so that regardless of mechanism, with appropriate measurement conditions, the event frequency can be used to determine the analyte concentration. The mechanism of **A1**-induced signal generation was investigated in a series of experiments. Using a setup (Supplementary Fig. 2) that physically separated electrodes and nanopore, events were only detected when **A1** was injected into the well proximal to the nanopore, thus supporting a signal generation mechanism involving interaction with the nanopore and not with the electrodes. This result did not, however, distinguish between passage-free collision with the nanopore opening (bumping or blocking) or translocation through the pore^32^. Either mechanism (including extending the idea of bumping or blocking to allow for transient interactions of the analyte with the pore mouth), though, has the potential to deliver analytically useful sensing performance. Low analyte concentrations challenge the direct investigation of polysaccharide translocation through small, single nanopores. In one experiment to investigate this, a solution of **A1** was added to the headstage side of a ~22 nm-diameter nanopore and was left overnight with a −200 mV voltage difference. The initially analyte-free contents of the ground-stage side were then transferred to the headstage side of a fresh ~17 nm-diameter pore, and an appreciable number of **A1**-characteristic events (182 in 1 h) were detected again using a −200 mV difference. Acid digestion was used as a signal generation and amplification technique (complete details in the Supplementary Methods) to convert **A1** polymers to many smaller fragment-derived species absorbing at ~270 nm^52,53^. This spectrophotometric assay (Supplementary Fig. 3) was used to confirm translocation of polysaccharide through an ~8 nm SiN_x_ nanopore.

**Fig. 3:**
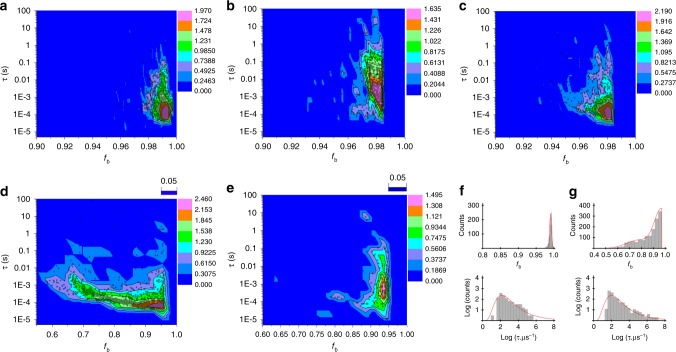
Heat maps with superimposed scatter plots of alginate-induced events. Event counts (plotted as log_10_ on the color axis) of **a** 4 µL 0.2% (w/v) **A1** using a ~19 nm diameter pore (~0.321 events·s^−1^), **b** 20 µL of 3% (w/v) **A2** using a ~21 nm (~0.046 events·s^−^^1^) and **c** 20 µL of 10-minute enzyme digested 3% (w/v) **A2** using a ~22 nm diameter pore (~0.112 events·s^−1^), all in pH ~7 buffered 1 M KCl. The experiment in **a** was repeated **d** using a ~5 nm nanopore (~0.403 events·s^−^^1^), and **e** an ~18 nm-diameter pore, but in 0.1 M KCl (vs. 1 M KCl in **a**) electrolyte buffered at pH ~7 (~0.0527 events·s^−^^1^). The plots in **f** and **g** show histograms of the data (the red lines show fits to the data) in **a** and **d**, respectively, with additional plots in Supplementary Fig. 4. The applied voltage difference was −200 mV for all measurements

The analyte-induced translocation blockage current, *i*_b_, is expected to be determined by the properties of the analyte and its size relative to the nanopore, among other experimental factors (including interfacial phenomena)^30,32^. For each individual current blockage, we calculated the blockage duration, *τ*, and the fractional blockage current magnitude, *f*_b_ = 〈*i*_b_〉/〈*i*_0_〉, where 〈···〉 denotes a time-average, and *i*_0_ is the current through the pore when unobstructed by analyte. Plots of number of events as a function of *τ* and *f*_b_ (Fig. 3) provide an overarching summary of the total current trace. Given detectable differences as a function of analyte, such plots and other representations have the potential to function as analyte fingerprints in quality assurance assays. Fingerprints for **A1** are shown in Fig. 3, acquired in 1 M KCl, pH ~7 solutions using a −200 mV voltage difference. Supplementary Figs. 4–6 provide alternative presentations of the experimental measurements. The (most frequent) *f*_b_ increased in magnitude with increasing nanopore radius, *r*_pore_ (that is, the relative magnitude of the current perturbations due to the analyte were reduced). This parallels the behavior observed in studies of DNA translocation that could be described using a simple volume-exclusion framework:$r_{{\mathrm{analyte}}}^2/r_{{\mathrm{pore}}}^2 = 1 - f_b$. While nanopore diameters are fixed once fabricated (absent etching), a conformationally flexible macromolecule can present a range of apparent cross-sections to a nanopore, down to its molecular cross-section if linearized by a sufficiently small nanopore. Translocation of hyaluronic acid through SiN_x_ nanopores fabricated using a helium ion microscope calibrated to produce ~6.5–8.6 nm-diameters yielded *f*_b_ ~ 0.95. Quasi-monodisperse samples gave single-level blockages with magnitudes of (1−*f*_b_), and multi-level blockages with magnitudes equal to (1−*f*_b_) and approximately twice that value, indicating the presence of linear and partially folded-over biopolymers, respectively^43^. Nanopore geometric constraints can thus affect the effective end-to-end length of the translocating molecule or, depending on the nature of the analyte, expose surface chemistry that can similarly affect translocation times. In Fig. 3d, the use of a ~5 nm-diameter nanopore broadened the distribution of *f*_b_ and produced deeper blockages with longer durations than when using the larger nanopore. Lowering the electrolyte concentration can have a dramatic effect on nanopore sensing, through changes in the bulk and at interfaces. For example, reducing the ion concentration from 1 to 0.1 M KCl increases the Debye layer thickness changing the electrostatic size of the pore with consequences for electrokinetic phenomena, and electroosmosis especially. Comparing Fig. 3a, e, this change of concentration did not affect the voltage polarity needed to generate events, but decreased the *f*_b_ for the same experimental configuration, and appreciably lengthened the (most frequent) blockage duration. More profoundly, the tenfold salt concentration decrease reduced the frequency of events sixfold in the same size ~18 nm-diameter pore. We found, and exploited in a more general context for the sensing of heparin and OSCS (below), that such a simple change of electrolyte concentration is a powerful parameter for tuning our ability to sense polysaccharides. Changing the electrolyte pH offers a similar parameter for tuning the sensing performance of nanopores with ionizable surface groups. The surface charge of SiN_x_ nanopores can be tuned from negative through its isoelectric point (~4.4 ± 0.3; standard deviation across three pores) to positive^45,54^, and the consequence of this pH change is seen in Supplementary Fig. 7: the voltage polarity for signal generation is opposite at pH 3 and 5 (and opposite to the electrophoretic direction for all pH values), and the event frequency is at its minimum nearest the isoelectric point and increases with increase and decrease in pH from this point.

After the initial exploratory and proof-of-principle experiments using *A1*, we turned to the second sodium alginate sample, **A2**, obtained from a separate supplier. In general, the interplay between analyte charge density, monomer chemical nature and polymer linkages, and electrolyte composition, is expected to influence nanopore sensing. Experiments showing the polarity-dependence of event occurrence, and its frequency, as a function of pH showed the same qualitative behavior as for **A1** in Supplementary Fig. 7, but with lower event frequencies overall. Both alginate samples were readily digested by alginate lyase (Supplementary Fig. 3)^55^, but infrared spectroscopy showed that **A2** contained a dramatically greater proportion of carboxylate groups than **A1** (Supplementary Fig. 8), so that the overall charge density of this molecule was expected to be higher than **A1**. Attempts to directly measure *ζ* potentials were complicated by corrosion of electrode surfaces, as noted in work examining the electrokinetics of protein transport through nanopores^56^. Further analysis was consistent with alginate **A1** having a ratio of guluoronic (G) to mannuronic (M) residues exceeding that of **A2**, with values from IR spectroscopy of ~63%G/37%M and ~57%G/43%M, respectively^49^. Nanopore profiling of **A2** showed differences compared to **A1**. Using the same electrolyte for **A2** as for **A1**, measurements generated a ~sevenfold lower event frequency with longer durations for **A2** compared to **A1**, in spite of the 75-fold higher **A2** concentrations required for reasonable measurement times. Enzymatic digestion of **A2** produced events at a higher frequency than for undigested **A2**, but still at lower frequency than for **A1**. The events for the digested sample of **A2** were tenfold shorter-lived than for the **A2** polymer, but not appreciably different in terms of blockage depth (Fig. 3, Supplementary Fig. 4). Measurements of the molecular-weight dependence of hyaluronic acid translocation through He-ion-drilled SiN_x_ nanopores showed >10–100-fold differences in event duration between ~50 and 2500 kDa species, where the differences were expected to arise from macromolecular size, alone. The attenuation of short-duration events due to instrument response characteristics has been detailed in earlier work, and is accommodated here through the use of suitable fitting functions^57,58^. The stand-alone reliability of extended measurements using the same ~10 nm pore for the alginate samples in series was limited by frequent partial pore clogging by **A2** that occasionally led to long-lived partial blockages that required cleaning steps that could change the apparent nanopore diameter by up to 3 nm. Nevertheless, these measurements using single pores yielded *f*_b_, *τ*, and event frequency characteristics for the three analytes consistent with the results in Fig. 3.

### Characterization of clinical and contaminated heparin

**Fig. 4:**
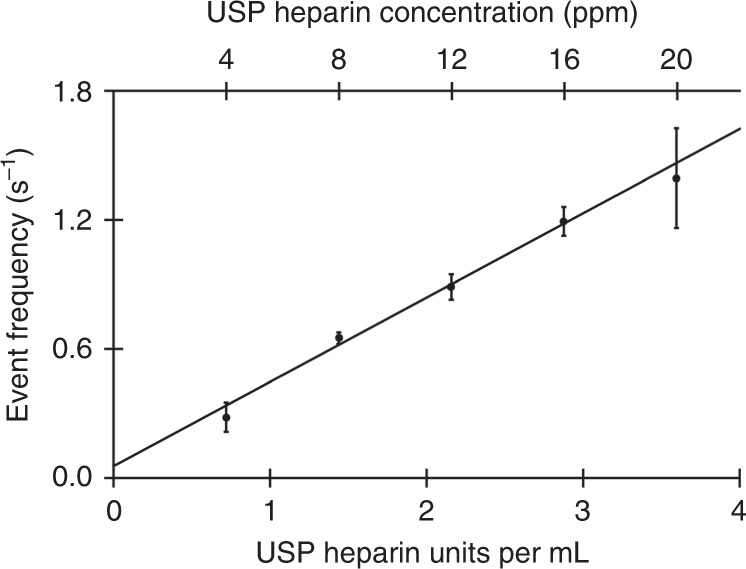
Heparin calibration curve. Three trials were performed, with at least 500 events per run extracted from 900 s-long measurements in a ~9.3 nm pore at +200 mV applied voltage difference after consecutive addition of 1 µL aliquots to the headstage side of the same nanopore. Error bars are the standard deviation for the three trials

**Fig. 5:**
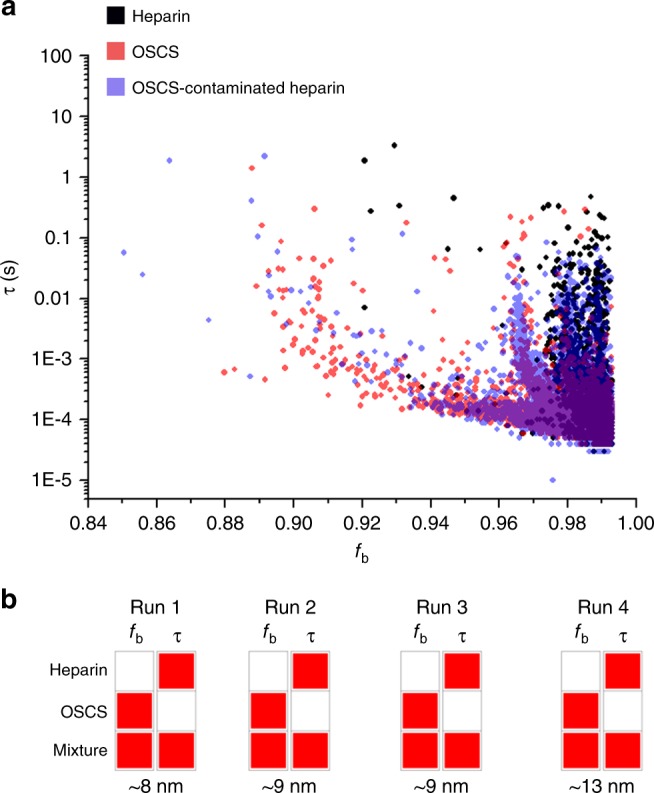
Nanopore resistive-pulse analysis of heparin, OSCS, and their mixture. **a** Superimposed scatter plots of 16 ppm heparin, OSCS and OSCS-contaminated heparin added to 4 M potassium chloride at +200 mV and measured using a ~13 nm pore. The colors in the legend correspond to the listed sample, and are blended (using transparency) in the plot where events from different samples overlap. **b** Recognition flags of heparin, OSCS and their mixture from four independent trials accurately identify the presence of the OSCS aliquot in the mixture. The red squares denote recognition of a species from the distribution of the corresponding property (column)

These initial survey experiments showed measurement outcomes with strong sensitivity to analyte identity, with the number of anionic carboxylate moieties being a compelling differentiator between **A1** and **A2**. We then turned to the pressing specific challenge of (anionic) heparin sensing and (anionic) OSCS impurity detection. The first change, from the earlier work, was that the signal generation voltage polarity (in 1 and 4 M KCl) now corresponded with the conventional electrophoretic direction for an anionic species. Acid digestion experiments akin to those in Supplementary Fig. 3 confirmed that heparin could translocate through the pore in response to an applied voltage. As with **A1**, heparin could be detected in 1 M KCl electrolyte, but the heparin event blockage magnitude and event frequency were both greater in 4 M KCl, and so measurements were performed at this higher salt concentration (see Supplementary Fig. 9 for representative events and a heat map). Plots of event frequency versus heparin concentration were linear (Fig. 4), with a limit of detection of 0.379 USP heparin units·mL^−1^ (in a 500 µL well). In comparison, clinical dosage levels of ~10^4^ units·day^−^^1^ using ~10^3^ units·mL^−^^1^ stock solutions are not uncommon. Heparin and alginate fingerprints differed in appearance from each other, but also through the profoundly different measurement configuration—opposite applied voltage polarity and fourfold higher electrolyte concentration for heparin—used to acquire them. We were more keenly interested, though, in whether an OSCS impurity in heparin could be detected. We performed measurements on unadulterated USP samples of either heparin or OSCS under identical experimental conditions. On the level of individual events, heparin and OSCS differed in their apparent interaction with the nanopore, with OSCS having a greater propensity to permanently block the pore unless a ~1.3 V (so-called zap) pulse—a common approach leveraging the electrokinetic basis of analyte motion—was quickly applied when indications suggesting an impending permanent blockage arose. In addition, events associated with the heparin and OSCS samples differed appreciably in the current fluctuations during individual current blockages: OSCS current blockages exhibited ~2–3 × greater current noise, *σ*(*f*_b_), than heparin-induced events. Overall, in spite of considerable overlap in the most frequent event *f*_b_ and *τ*, the distribution of event characteristics revealed a key difference between heparin and OSCS samples (Fig. 5 and Supplementary Fig. 10). Namely, events measured using heparin samples exhibited a longer duration tail in the total event duration distribution, while events measured using OSCS samples exhibited a longer tail in *f*_b_. Measurements of mixtures of heparin and OSCS (16 ppm each) yielded event distributions showing both tails, consistent with the presence of both the heparin therapeutic and its contaminant. We developed an automatic thresholding procedure based on event distribution statistics in *f*_b_ and *τ* (details in the Supplementary Methods) to collapse the event distribution fingerprints into recognition flags denoting the presence or absence of each component. In brief, OSCS was declared present when events occurred with $f_{{\mathrm{b,sample}}} \lesssim {\mathrm{mode}}({f_{{\mathrm{b,USP}}\,{\mathrm{heparin}}}^{{\mathrm{binned}}}}) - 3\sigma ({f_{{\mathrm{b,USP}}\,{\mathrm{heparin}}}^{{\mathrm{binned}}}})$ and heparin was declared present when events occurred with $\tau _{{\mathrm{sample}}} \gtrsim {\mathrm{mode}} ( ( {\log}_{10}\tau _{{\mathrm{USP}}\,{\mathrm{OSCS}}} )^{{\mathrm{binned}}} ) - 3\sigma ( ( {\log} _{10}{\tau }_{{\mathrm{USP}}\,{\mathrm{OSCS}}} )^{{\mathrm{binned}}})$. Figure 5 shows the correct recognition of USP heparin, USP OSCS, and a mixture of both, across four trials using nanopores of slightly different sizes. The OSCS contaminant levels detected here were fourfold lower (without efforts to explore a lower bound) than the OSCS detection limit reported in the work that examined and quantified the contaminant in suspect heparin lots^18^.

## Discussion

We demonstrated the feasibility of using SiN_x_ nanopores to characterize glycans exhibiting a variety of chemical compositions, including a prevalent therapeutic, heparin. The extremely high-charge density carried by heparin poses a particular challenge to a nanoscale sensor element that can, itself, be charged. More generally, unwanted interactions between analyte and nanopore—and the ease and feasibility of ameliorative steps—can imperil nanopore-based experiments: that none of the diverse polysaccharides considered here catastrophically clogged the nanopore—even when subjected to the stringent test of translocation through the pore–was salutary^48^. Indeed, nanopore sensing was successful over a number of electrolyte concentration ranges, from 0.1 to 4 M KCl, for which shielding of the charged nanopore surface would be quite different in degree. With translocation possible through SiN_x_ nanopores, even with their charged surface, a rich set of nanopore-based sensing configurations should be within reach. In this work, we used a straightforward resistive-pulse sensing paradigm to readily detect and differentiate between different polysaccharides, including enzymatic digestion products and two separate alginate samples differing in relative monomer composition. We used voltage polarity and electrolyte composition alongside the distribution of events as a function of *f*_b_ and *τ* to construct fingerprints and recognition flags characteristic of each sample. Linear calibration curves show that these measurements easily support concentration determinations in addition to analyte recognition.

From a fundamental perspective, nanopores can be a powerful tool for exploring molecular, interfacial, and intermolecular phenomena, often arising from only simple changes of experimental conditions. Electrolyte-dependent interfacial interactions—at nanopore and molecule surfaces—are complex, and treatments of widely varying levels of sophistication have emerged from decades of experimental and theoretical studies of the canonical nanopore-DNA system, in particular^32^. For example, changes of electrolyte concentration have been observed to reverse the sign of the current perturbation in DNA translocations through solid-state nanopores, and to decrease dextran sulfate blockage frequencies while increasing their durations using ~1.3 nm-diameter pores where the Debye length was comparable to the pore dimensions^42,59^. With the larger pores used here, overlapping Debye layers would not be expected in 0.1 M KCl solutions, leaving three expected principal effects of lowering the electrolyte concentration from 1 M KCl: a lowering of the potential across the pore and thus of the overall electrophoretic force on an analyte near the pore; a reduction in the available number of bulk ions displaced by the analyte volume; and a change in the ion distribution around charged interfaces—the nanopore and analyte surfaces—that influences the nanopore signal through a complex overall mechanism within a given experimental configuration. Blockage magnitudes measured here in the more conventional 1 M KCl would be consistent with, in a simple volume exclusion sense ($r_{{\mathrm{analyte}}}^2/r_{{\mathrm{pore}}}^2 = 1 - f_b$), translocation of linearized polysaccharides. Deeper blockages would be expected from the polysaccharides here with hydrodynamic radii on par with the nanopore diameters (for example, **A1** and **A2** have viscosity-derived free-solution hydrodynamic radii of ~19 and ~8 nm). Polysaccharide translocation was independently confirmed and signals were detected only when the analytes had access to the nanopores, so these recorded events either arose from analyte interactions with the pore mouth rather than from translocation, or the blockage magnitude analysis must include additional factors such as charge density carried by the analyte, itself, and consider mobile charges at the analyte-solution and solution-nanopore interfaces^59,60^. The effects of these and more complex interfacial phenomena emerged in one of the more unexpected observations in this work: that the voltage polarity for signal generation with both alginate samples was opposite to that expected for electrophoretic motion of an anionic polymer (for example, hyaluronic acid in He-ion-drilled SiN_x_ pores^43^), and the more charge-rich **A2** was detected at a lower event frequency than **A1**. The voltage polarity contrasted with the electrophoretic polarity for detecting highly charge-dense, anionic heparin. Nanopore-based studies with polyethylene glycol polymers point to a change of effective analyte charge by sorption of electrolyte ions (K^+^ for those studies) with the resultant analyte motion then being electrophoretic for the voltage polarity and the sign of the sorbed charge^29^. The results of Supplementary Fig. 7, however, point to pH-dependent changes in the voltage polarity required for sensing alginates, with the polarity having opposite signs on either side of the isoelectric point of SiN_x_. Mirroring this change in the voltage polarity is the SiN_x_ surface charge that is positive at lower pH and negative at higher pH^45,46,56^. This change in nanopore surface charge sign causes a reversal in the direction of electroosmotic motion for a fixed voltage polarity (and thus fixed electrophoretic direction). In addition, when an analyte contains at least one ionizable moiety (with associated pK_a_), then changes of solution pH can also affect the analyte charge sign and density—and thus the voltage polarity required for electrophoresis in a given direction. The apparent mobility of an analyte in response to electrolyte flow through the surface-charged nanochannel is the sum of its electrophoretic and electroosmotic mobilities, which can both be tuned by the solution pH. Work examining the electrokinetics of protein transport through silicon nitride nanopores showed that electroosmosis could overwhelm electrophoresis as the effect determining the direction of analyte motion^56^. Given the acidic functional groups in the analytes here, the changes in nanopore surface chemistry should dominate the effective mobility and its voltage polarity dependence. The event frequency and voltage polarity behaviors are consistent with the distinct physicochemical properties of each analyte with both electrophoresis and electroosmosis occuring simultaneously. In the negatively charged SiN_x_ pores at pH~7, electroosmosis and electrophoresis are in opposition for anionic **A1** and **A2**, and signal was generated in the electroosmotic direction for both. The electrophoretic force would be greater on the more highly charged **A2**, lowering its detection frequency in the opposing electroosmotic direction, relative to **A1**, consistent with observation. More detailed exploration of the differences between **A1** and **A2** must also contend with their different molecular weights and their different chain flexibilities arising from their different M/G ratios. In the case of heparin, the charge density is sufficiently high so that events are detected using a voltage polarity that would electrophoretically drive the anionic polymer towards the nanopore. Experimental investigations including and beyond the ones presented here, exploring the underpinnings of the nanopore-generated signal using (polysaccharide) biopolymers such as these, with greater chemical and structural complexity than the canonical nanopore test molecule, DNA, or than homopolymers such as polyethylene glycol, should also provide fertile ground for high-level simulations. Interfacial effects will require additional study in the context of polysaccharides, but hold possibilities for tuning sensing selectivity and sensitivity. Indeed, explicit consideration of sensing conditions—including nanopore size, electrolyte composition, and voltage polarity—already augments the ability to compare nanopore molecular fingerprints as shown in Fig. 3.

The failure in 2008 to detect an OSCS contaminant in clinical heparin samples had previously led to patient morbidity and mortality^14–18^, so that our ability to use a simple nanopore-based assay to quantify heparin levels and detect OSCS at clinically meaningful contamination levels, is itself significant. In a broader sense, we expect that these initial results exploring polysaccharide structure can, by analogy with earlier nanopore DNA and protein sensing supporting genomics and proteomics, spotlight the potential of using nanopores as a tool for glycomics. The demonstration of polysaccharide translocation through nanofabrication-compatible SiN_x_ nanopores portends the development of more sophisticated sensing schemes as seen in the use of nanopores for genomics. Similarly, the successful use of chemical tuning—of electrolyte composition and by enzyme addition—to alter the nanopore signal generated by diverse polysaccharides suggests that nanopore glycomics might borrow from and extend upon similar approaches developed for nanopore genomics. There is an ongoing need in glycomics for new tools to cope with the analytical challenges caused by the structural and physicochemical complexity of polysaccharides, and by the often inherently heterogenous nature of naturally derived carbohydrates. The demonstrations of nanopore sensing here provide a beachhead for ongoing efforts to develop solid-state nanopores as a promising platform technology for glycomics.

## Methods

### Nanopore formation and characterization

A full listing of the experimental details is available in the Supplementary Information. Nanopores were formed via controlled dielectric breakdown^44^ in nominally 10 nm-thick silicon nitride (SiN_x_) membranes. Apparent nanopore sizes were inferred from their conductance, *G*, determined from Ohmic current-voltage data. This method for determining the size of a single nanopore has long-standing use, but has gained greater prominence with the emergence of low-overhead nanopore fabrication methods such as various implementations of the dielectric breakdown method^44,61,62^. Conductance-based characterization provides effective characterization of nanopores without the burdens of charged-particle microscope use, and so we adopted the approach within its conventional application framework.^44–46,58,59,63–67^. Nanopores used for measurements produced stable open-pore (analyte-free) currents in the electrolyte solutions used.

### Polysaccharide profiling

Polysaccharides were commercially obtained: sodium alginate samples from two different sources—**A1** (Alfa Aesar, Ward Hill, MA) and **A2** (FMC Corporation Health and Nutrition, PA, USA); USP heparin sodium salt; and USP OSCS. For routine measurements, sample aliquots were added to the headstage side (Fig. 1), leaving the ground side free of initially added analyte. Current blockages were extracted using a current-threshold analysis. The experiment was configured as shown in Fig. 1, and applied voltage differences ∆*V* = −*V*_headstage electrode_ were reported so that a positive value would be required for electrophoretic passage of an anionic molecule through the nanopore.

### Data availability

The authors declare that the data supporting the findings of this study are available within the paper and its Supplementary Information files.

## Electronic supplementary material

Supplementary Information
