# Supplementary material for: Surveying silicon nitride nanopores for glycomics and heparin quality assurance
Source: Nat Commun. 2018 Aug 16;9:3278. doi: 10.1038/s41467-018-05751-y (PMC6095881; doi:10.1038/s41467-018-05751-y)
Supplement: Supplementary file 1 — Supplementary Information [file 41467_2018_5751_MOESM1_ESM.pdf]

Supplementary Information

Surveying Silicon Nitride Nanopores for Glycomics and Heparin Quality Assurance

Karawdeniya *et al.*

SUPPLEMENTARY METHODS

Reagents and Materials.

The following materials, identified by their product number and specification, were obtained from Sigma-Aldrich Corporation (St. Louis, MO, USA): potassium chloride (60130, puriss. p.a., ≥99.5% (AT)); sodium chloride (S7653, BioXtra, ≥99.5% (AT)); HEPES potassium salt (H0527, ≥99.5% (titration)); sulphuric acid (339741, 99.999%); alginate lyase (A1603, ≥10,000 units$\cdot g^{-1}$); and hydrochloric acid (320331, ACS reagent, 37%). Polysaccharides were commercially obtained: sodium alginate **A1**-B25266 (~75-120 kDa, 40-90 centipoise (1% solution); Alfa Aesar [Ward Hill, MA, USA]) and **A2**- PROTANAL® LFR5/60 (120kDa, 300-700 centipoise (10% solution); FMC Corporation Health and Nutrition, PA, USA); heparin sodium salt (USP, 1304038, Rockville, MD; mol. wt. ~16 kDa by lot certificate) and over sulfated chondroitin sulfate (OSCS) (USP, 1133580; est. mol. wt. ~17 kDa by porcine origin[1]; from Sigma Aldrich Corporation (St. Louis, MO, USA)). The potency of the USP heparin samples was 180 USP heparin units according to Pharmacopeial Forum Vol. 35(5) [Sept.–Oct. 2009].

Silicon-rich LPCVD silicon nitride (nominally) 10 nm-thick membranes on 200 µm-thick silicon frame (NT001Z and NT005Z; with reported membrane thicknesses (±manufacturer-supplied tolerance) for Lot # L8 10.5±0.3 nm, L15 16±2 nm, L31 14±2 nm, L68 10±1.5 nm) were purchased from Norcada, Inc. (Alberta, Canada).

All aqueous solutions were prepared using Type I water (~18 MΩ·cm resistivity from either a Millipore Synergy UV [Billerica, MA], or American Aqua Maxicab system [Narragansett, RI, USA]); all dilutions and washes also used this water. Stericup-VP vacuum filtration systems were used to filter electrolyte solutions after preparation, and water to prepare alginate solutions (SCVPU11RE 0.10 µm pore size in polyethersulfone membrane; EMD Millipore Corporation [MA, USA]).

Ag/AgCl electrodes were made from 1.0 mm-diameter silver wire (Alfa Aesar 11434, annealed, 99.9% (metals basis)) by soaking overnight in sodium hypochlorite (Alfa Aesar 33369, 11-15% available chlorine). Electrodes were insulated using shrink-wrap PTFE tubing (McMaster-Carr, 7960K21, high-temperature harsh environment tubing, moisture seal, heat-shrink, 0.07" ID before; and 7564K67, high-temperature harsh environment tubing, heat-shrink, 0.08" ID before, 0.05" ID after) and connected to electronics using pins (Connectivity TE Connectivity / AMP 205090-1 D sub circular connector contact, AMPLIMITE 109 Series, Socket, Crimp, 20-24 AWG). Nanopore chips were compressed between silicone gaskets (McMaster-Carr, 86435K43, high-temperature silicone rubber sheet, ultra-thin, 12" x 12", 0.015" thick, 35A durometer) in custom-machined PTFE holders with ~500 µL sample wells.[2]

Silicone tubing with ID 1.0 mm x OD 3.0 mm was obtained from Nanion Technologies GmbH, Munich, Germany.

Instrumental Details.

Measurements of solution pH and conductivity were with an Orion Star™ pH meter and Orion™ ROSS Ultra™ Refillable pH/ATC Triode™ Combination Electrodes and Orion™ DuraProbe™ 4-Electrode Conductivity Cells (Thermo Fisher Scientific Inc, MA, USA).

Nanopore formation by dielectric breakdown was performed using programmable DC power supplies (Model 9121A, B&K Precision Corporation, CA, USA) interfaced to a home-built circuit;[3] real-time current measurements were by a 428-Programmable Current Amplifier (Keithley Instruments, Cleveland, OH, USA) interfaced to NI USB 6351 DAQ card using custom LabView-based (National Instruments Corp., TX, USA) software to control the applied voltage.

All nanopore measurements were performed using an Axopatch 200B amplifier (Axon Instruments, Foster City, CA, USA) in voltage clamp mode. The amplifier was interfaced to a computer system using a data acquisition card (779512-01 NI PCIE-6251 M Series with 777960-01 NI BNC-2120 shielded connector block) and control software written in LabView. Current-versus-time measurements were typically acquired for 1 h (3× 20 min) at 100 kHz acquisition rates with the 4-pole low pass Bessel filter built-in to the Axopatch 200B set to 10 kHz. Measurements of nanopore conductance were acquired at a rate of 10 kHz, with the filter set to 1 kHz.

Infrared spectra of the powder were acquired by FTIR-ATR (Bruker Tensor 27 equipped with a Ge crystal) averaged over 256 scans with 4 cm^{-1} spectral resolution. All measurements done inside a nitrogen filled glovebox.

UV/Vis spectra were collected using a Varian Cary 50 Bio UV/Visible Spectrophotometer with a quartz cuvette with a 1 cm pathlength. Single run measurements were taken from 200 to 400 nm at a scan rate of 300 $nm \cdot min^{-1}$ and 0.50 nm intervals.

All 3D printed components were designed in Solid Works 2014 Professional Edition (Dassault Systems SolidWorks Corporation, Waltham, MA) and printed by Makerbot Replicator (MakerBot Industries, Brooklyn, NY) using PLA plastic (MP06103, MakerBot Industries, Brooklyn, NY).

General Nanopore Sensing Procedure.

Nanopores in the ~10 nm-thick silicon nitride membranes were fabricated by controlled dielectric breakdown using 11-15.5 V DC applied potentials.[3] The nanopore formation was carried out in 1 M KCl electrolyte, HEPES-buffered to pH ~7, and the membranes and pores were secured in custom-machined PTFE holders with ~500 µL sample wells.

Nanopore conductances, G, were the slope of the linear fit to the experimental Ohmic current-voltage data, measured in 1 M KCl electrolyte buffered with HEPES at pH ~7. The corresponding nominal nanopore diameters were calculated using a conductance model (including bulk, surface, and access resistance terms) and cylindrical nanopore shape suitable for this salt concentration and fabrication method,[3, 4, 5, 6]

$$G = \left(\frac{1}{G_{\text{bulk}} + G_{\text{surface}}} + \frac{1}{G_{\text{access}}}\right)^{-1}. \tag{1}$$

Nanopores used for measurements produced stable open-pore (analyte-free) currents at the salt concentrations used.

All electrolyte solutions were HEPES-buffered (10 mM) to pH ~7 unless otherwise noted (adjusted with dropwise addition of concentrated hydrochloric acid), and measurements were carried out using filtered solutions with 0.1, 1.0, and 4.0 M KCl concentrations. Solutions of 0.2% (w/v) sodium alginate, 0.2% (w/v) heparin, and 0.2% (w/v) OSCS were made by dissolving the solids in filtered Type I water. For routine

measurements and unless otherwise specified, 4 μL aliquots were added to the headstage side (Figure 1), leaving the ground side free of initially added analyte. Calibration curves for each nanopore were constructed by repeated cycles of measurement followed by the addition of another analyte aliquot. Current blockages were extracted using a current-threshold analysis. Any current blockages exceeding 100 s ($\lesssim$ 0.1%) were not included in analyses.

Polysaccharide Viscosity Measurements.

Apparent viscosity measurements were carried out on aqueous sodium alginate solutions (0.15-1.0 $g \cdot dL^{-1}$) in 0.1 M sodium chloride solutions using a capillary viscometer (SI Analytics Ubbelohde Viscometer, Thermo Fisher Scientific, Inc., MA, USA) immersed in a water bath at ~23°C. Triplicate measurements of the apparent viscosity were made at each solution concentration to yield the intrinsic viscosity, $[\eta]$, from a plot of[7]

$$\frac{\eta_{\mathrm{sp}}}{C} = [\eta] + k[\eta]^2 C \tag{2}$$

where C is the macromolecule's concentration in $g \cdot dL^{-1}$, k is a constant characteristic of the solute-solvent system, $\eta_{\mathrm{sp}} = \frac{\eta_{\mathrm{solution}}}{\eta_{\mathrm{solvent}}} - 1$ is the specific viscosity calculated from the apparent viscosities. The weight- and number-average molecular masses, M_{w} and M_{n}, and the of the polymers in kDa were calculated according to[8]

$$[\eta] = 0.023(M_{\mathrm{w}})^{0.984} \tag{3}$$

$$[\eta] = 0.095(M_{\mathrm{n}})^{0.963}. \tag{4}$$

The respective molecular masses of the two alginate samples were determined by this method to be ~286 kDa and ~74 kDa for **A1**, and ~71 kDa and ~18 kDa for **A2**. Using a polymer's molecular weight, M, we can calculate the hydrodynamic radius (N_{A} is Avogadro's number)[9]

$$R_{\mathrm{h}} = \left(\frac{3[\eta]M}{10\pi N_{\mathrm{A}}}\right)^{1/3} \tag{5}$$

to be ~19 nm for **A1** and ~8 nm for **A2** (on an M_{n}-basis). The corresponding root-mean-squared end-to-end distance, $\langle \overline{r^2} \rangle^{1/2}$ for each sample is equal to $3.1 R_{\mathrm{h}}$.

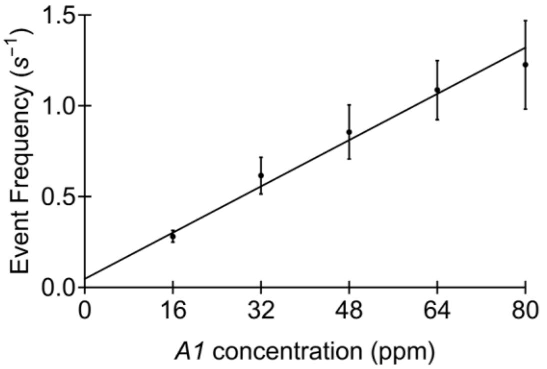

Supplementary Figure 1: Calibration curve of **A1** event frequency versus concentration of **A1**. Three trials were performed, with each data point including at least 1000 events extracted from at least 1 h long measurements using a -200 mV applied voltage difference after consecutive additions of 4 µL aliquots to the headstage side of the same nanopore. Error bars represent the standard deviation across the three trials.

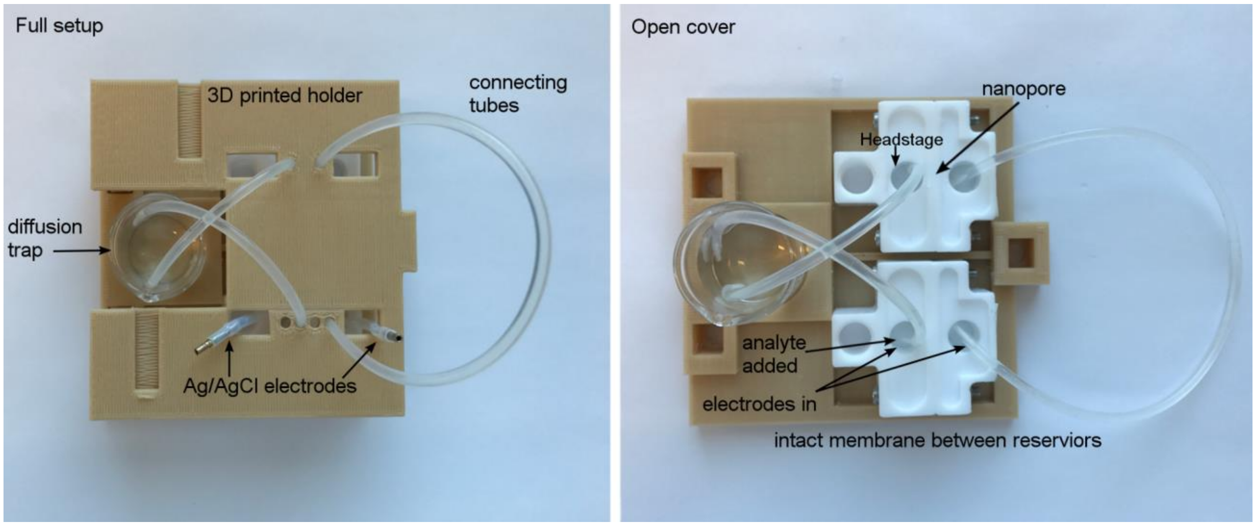

Supplementary Figure 2: A special nanopore configuration in which the electrolyte wells proximal to the electrodes and to the nanopore were physically separated. The purpose of this configuration was to determine if the current blockages arose from analyte interaction with the electrodes, or with the nanopore, itself. The electrolyte wells in the lower PTFE cell held the electrodes and were separated by an intact SiN_x membrane that did not allow ionic flow. These wells were connected through electrolyte-filled silicone tubing and an electrolyte-filled beaker (acting as a diffusion trap), to a second electrolyte-filled PTFE cell in which the wells were separated by a SiN_x nanopore. With analyte injected into the bottom cell, the only possible mechanism of current blockage was either by direct interaction with the electrodes, or by the passage of analyte through the tubing and beaker of solution until it could interact with the nanopore. When a 4 µL aliquot of the alginate was added to the headstage side of the lower cell, only 18 appreciable current transients were detected in a 1 hour measuring period, contrasted with 561 events in 1 hour when the alginate was directly injected adjacent to the headstage side of the nanopore, both measurements using a -200 mV voltage difference. The additional electrolyte between electrodes and nanopore reduces the cross-pore applied potential compared to the usual single-cell sensing configuration.

Acid and Enzymatic Digestion Procedures.

Acid digestion post-nanopore measurement. An ~8 nm nanopore was mounted in the PTFE sample holder. A 200 µL amount of 0.2% (w/v) **A1** was added to the headstage side in 5 µL aliquots per hour throughout the work day during 4 days of application of a -200 mV cross-membrane voltage. For overnight voltage applications, the electrode polarity was reversed. The headstage and initially analyte-free ground side solutions were extracted, individually mixed with 1 mL of 75% sulphuric acid and heated overnight (16 h) at 80°C. Samples were diluted with 3 mL of water before spectral acquisition. For comparison, 500 µL aliquots of 0.2% (w/v) **A1** and **A2** were each subjected to the same acid digestion and dilution before spectral acquisition.

Enzymatic digestion for spectroscopic measurements. A 2250 µL aliquot of 0.2% (w/v) **A1** was added to a 150 µL aliquot of 1 unit·mL^{-1} alginate lyase and heated in a water bath at 37˚C for 30 minutes. The procedure was repeated for sample **A2**, but the sample was diluted with 10 mL H_2O before spectral acquisition.

Enzymatic sample preparation for nanopore sensing. For enzymatic digestion, samples of 3% (w/v) **A2** were mixed with alginate lyase (1:1 (v/v) mixture with 1 unit·mL^{-1} enzyme) for 10 minutes at 37°C. 20 µL of this mixture was added to the headstage side and events were detected with the application of -200 mV on the headstage side. Measurements in the presence of 20 µL of 1 unit·mL^{-1} of alginate lyase, alone, in the

headstage side support that the detected events in the presence of analyte originated from enzymatic digestion products.

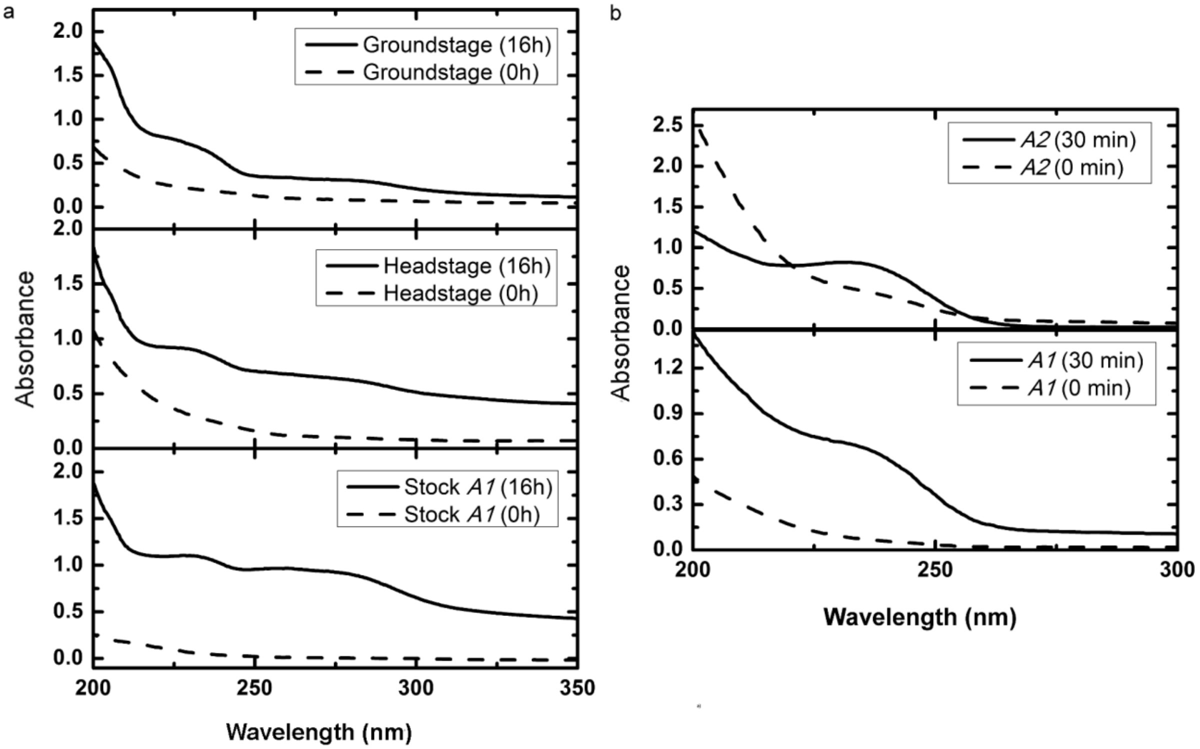

Supplementary Figure 3. UV/Vis spectra of acid and enzymatic digestion products. a) Stock **A1** subjected to 16 h of sulphuric acid digestion generated a ~270 nm absorption band characteristic of the digested polysaccharide[10, 11] that was replicated in the samples taken from the headstage and from the ground-side sample wells after 4 days of a translocation experiment (200 µL aliquot). The dashed lines denote the UV/Vis spectra of the sample before digestion, and the solid lines denote the spectra after digestion. b) Alginate lyase digestion of alginate is expected to introduce chromophores with a peak absorption at ~232 nm, consistent with observations here.[12]

Preparation of Heat Maps by Histogramming Individual Events.

Heat maps were prepared in Origin (Originlab Corporation, MA) from event data sorted into bins by paired f_b and τ. The bin width along the f_b axis was set equal to $W_\mathrm{bin} = 3.49\sigma(f_\mathrm{b})N^{-\frac{1}{3}}$, where $\sigma(f_\mathrm{b})$ is the standard deviation across all events, and N is the total number of events.[13] Bin size along the τ axis was set to $\sqrt{10}$. Heat maps are plotted using $\log_{10}$ of the number of events in each bin.

The distributions of event counts by f_b in Supplementary Figure 4 (top row) were fit using the function

$$\phi_{f_\mathrm{b}} = \frac{1}{2}(1+\theta)\sum_{i=1}^{M} A_i \cdot \exp\left(-\frac{(f_\mathrm{b}-\mu_i)^2}{2\sigma_i^2}\right) \quad (6)$$

where the parameters of the unmodified Gaussian function are as conventional: A_i, μ_i, and σ_i are the magnitude scaling, expected value, and standard deviation. The step function, $(1+\theta)$, was set to 1 for$f_\mathrm{b} < f_\mathrm{b}^\mathrm{cutoff} + W_\mathrm{bin}$, and 0 otherwise, so that the fit function covers only the accessible experimental data ($f_\mathrm{b}^\mathrm{cutoff}$ was the threshold for event extraction). The best-fit parameter values are shown in Supplementary Table 1.

Panel	A_1	μ_1	σ_1
a	364<br>A_2 =76	0.971<br>μ_2 =0.773	0.0624<br>σ_2 =0.0992
b	240	0.991	0.00274
c	150	0.98	0.00558
d	100<br>A_2=304	0.974<br>μ_2 =0.979	0.0041<br>σ_2 =0.002
e	312	0.991	0.00635
f	500<br>A_2=2120	0.985<br>μ_2 = 0.989	0.0077<br>σ_2 =0.0016

Supplementary Table 1: Best-fit parameters for ϕ_{f_b} used in Supplementary Figure 4 (top row).

The distributions of the log of event counts by duration (middle row of Supplementary Figure 4) were fit to a log-normal distribution[14]

$$\phi_\tau = \frac{A}{\tau} e^{-(\ln\tau - M)^2/(2S^2)} \tag{7}$$

where the parameters had conventional meanings, and the event duration was expressed in µs. The event duration corresponding to the peak of the event count distribution, τ_p, was found by taking the first derivative of the curve. The best-fit parameter values are shown in Supplementary Table 2.

Panel	A	M	S	τ_p (µs)
a	5.49	1.01	0.57	98.91
b	5.93	1.07	0.55	143.98
c	6.95	1.38	0.51	1102.32
d	5.43	1.11	0.67	89.31
e	6.62	1.15	0.55	218.69
f	6.85	0.81	0.50	57.27

Supplementary Table 2: Best-fit parameters for ϕ_τ used in Supplementary Figure 4 (middle row).

The distributions of the event counts versus event duration were fit, over the temporal range shown in the bottom row of Supplementary Figure 4, to a sum of exponential decays[15, 16, 17] with a step function cutoff convolved with a Gaussian distribution,

$$\phi_\tau^{\mathrm{exp}} = \exp\left({}^{-\tau}/{}_{2\sigma^2}\right) * \left(\left(A_1 \exp(-\tau/\tau_1) + A_2 \exp(-\tau/\tau_2)\right) \cdot \theta\right). \qquad (8)$$

The step function, θ truncated the decay functions below the histogram modes, τ_{mode}, and all listed parameters were unconstrained during fitting using the NMinimize method of the NonlinearModelFit of Mathematica 11.0.1.0. The peak time, τ_{p}, is taken as the peak position in ordinate. The best-fit parameter values are shown in Supplementary Table 3.

Panel	σ	A_1	$\tau_1(\mu S)$	A_2	$\tau_2(\mu S)$	$\tau_{\mathrm{mode}}(\mu S)$	$\tau_{\mathrm{p}}(\mu S)$
a	11.88	5648.42	8.9	3.11	113.82	50	58.31
b	34.0	17339.1	10.99	0.25	298.06	90	103.63
c	1.00	117.92	49.75	7.93	2052.88	100	102.62
d	25.92	10021.6	11.79	0.68	193.56	90	104.38
e	16.98	114119	6.81	0.99	227.27	60	67.86
f	11.56	51000.2	10.84	12.10	103.92	60	68.17

Supplementary Table 3: Best-fit parameters for ϕ_τ^{exp} used in Supplementary Figure 4 (bottom row).

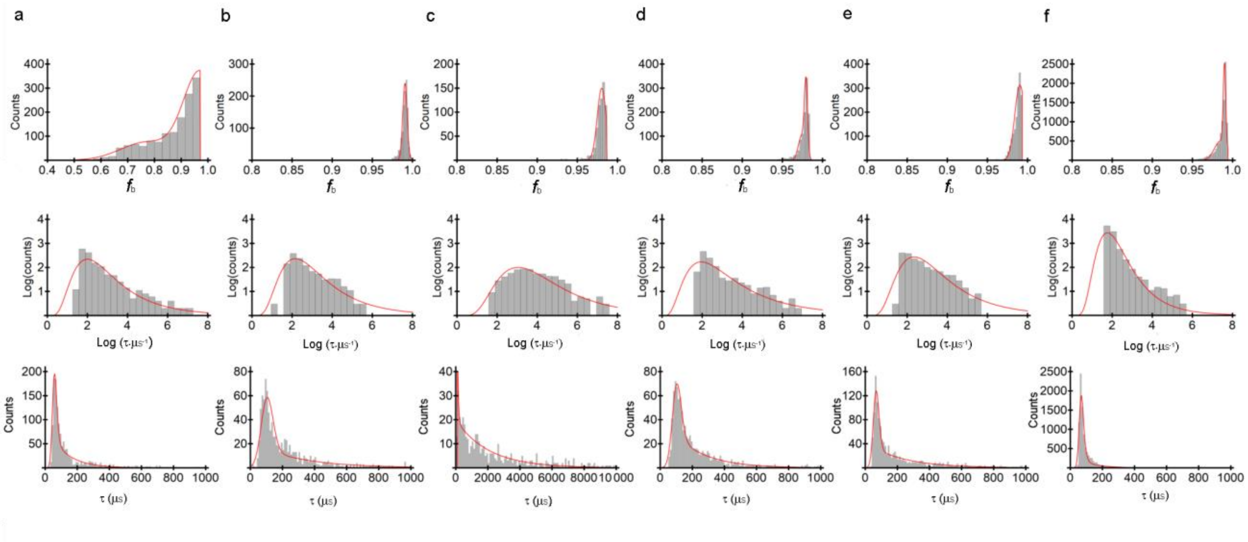

Supplementary Figure 4: Histograms describing alginate event characteristics. Histograms of (top row) f_{b} (middle & bottom rows—log normal and exponential fits over the displayed temporal range, respectively) duration in $\log_{10}$ of **A1** alginate in (a) ~5 nm and (b) ~19 nm pore, **A2** in (c) ~21 nm, (d) 10-min enzyme digested **A2** in ~22 nm pore, (e) heparin and (f) OSCS in the same ~13 nm pore with the bin size set automatically by the measurement statistics as described above. The red line in each histogram represents the fit using the specific function noted.

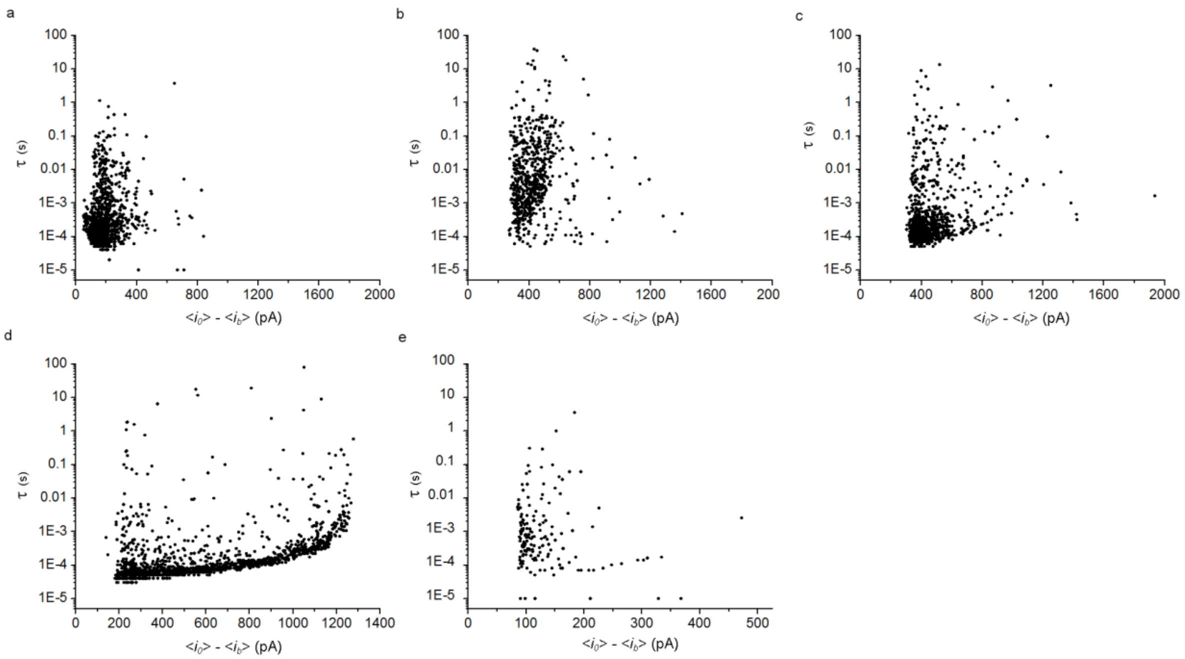

Supplementary Figure 5: Scatter plots of alginate-induced events from Figure 3, plotted against $\langle i_b \rangle - \langle i_0 \rangle$ instead of f_b. Events corresponding to (a) 4 µL 0.2% (w/v) **A1** using a ~19 nm diameter pore (~0.321 events·s^{-1}), (b) 20 µL of 3% (w/v) **A2** using a ~21 nm (~0.046 events·s^{-1}) and (c) 20 µL of 10-minute enzyme digested 3% (w/v) **A2** using a ~22 nm diameter pore (~0.112 events·s^{-1}), all in pH ~7 buffered 1 M KCl. The experiment in (a) was repeated (d) using a ~5 nm nanopore (~0.403 events·s^{-1}), and (e) an ~18 nm-diameter pore, but in 0.1 M KCl (vs. 1M KCl in (a)) electrolyte buffered at pH ~7 (~0.0527 events·s^{-1}). The applied voltage difference was -200mV for all measurements.

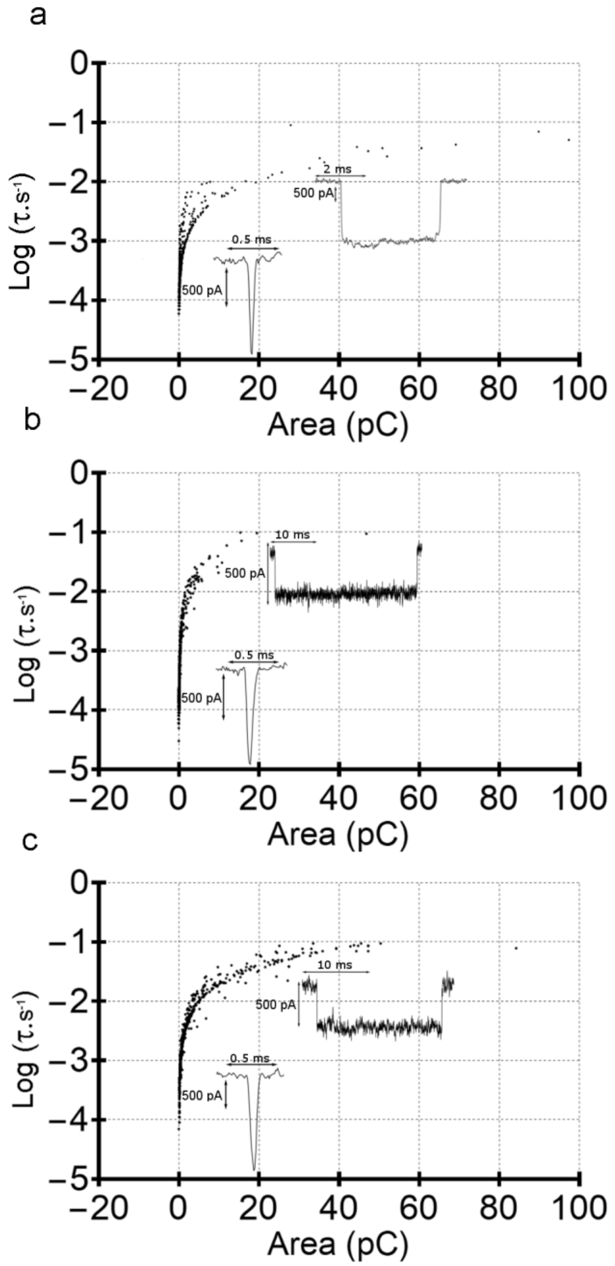

Supplementary Figure 6: Analysis of nanopore events by duration and charge. Plots of $\log_{10}$ of event duration (τ) versus area under each event for alginate **A1** in (a) ~5 nm and (b) ~19 nm diameter pores and c) for alginate **A2** in a ~21 nm diameter pore recorded for 1 hour in 1 M KCl at pH ~7. Two distinct event distribution tails are visible corresponding to short-lived spike-like pulses and longer-lived rectangular blockages. The longer-lived tail for **A2** is more prominent as a percentage of total events than for **A1**, consistent with the appearance of the combined heat and scatter plots in Figure 3. The shorter events could be attributed to either "bumps" or fast translocations, and longer-lived events could be attributed to slower translocations or longer-lived interactions with the pore (in both cases, complementary measurements independently confirmed that alginate was able to translocate through the nanopores). The low molecular weight of **A2** and stiffness associated with its high M/G ratio[18, 19] suggest a greater likelihood of translocating through the pore that could explain the longer tails for **A2** compared to **A1**. The area under each event was calculated by integrating the interpolation function (interpolation order of 1) of each event in Mathematica.

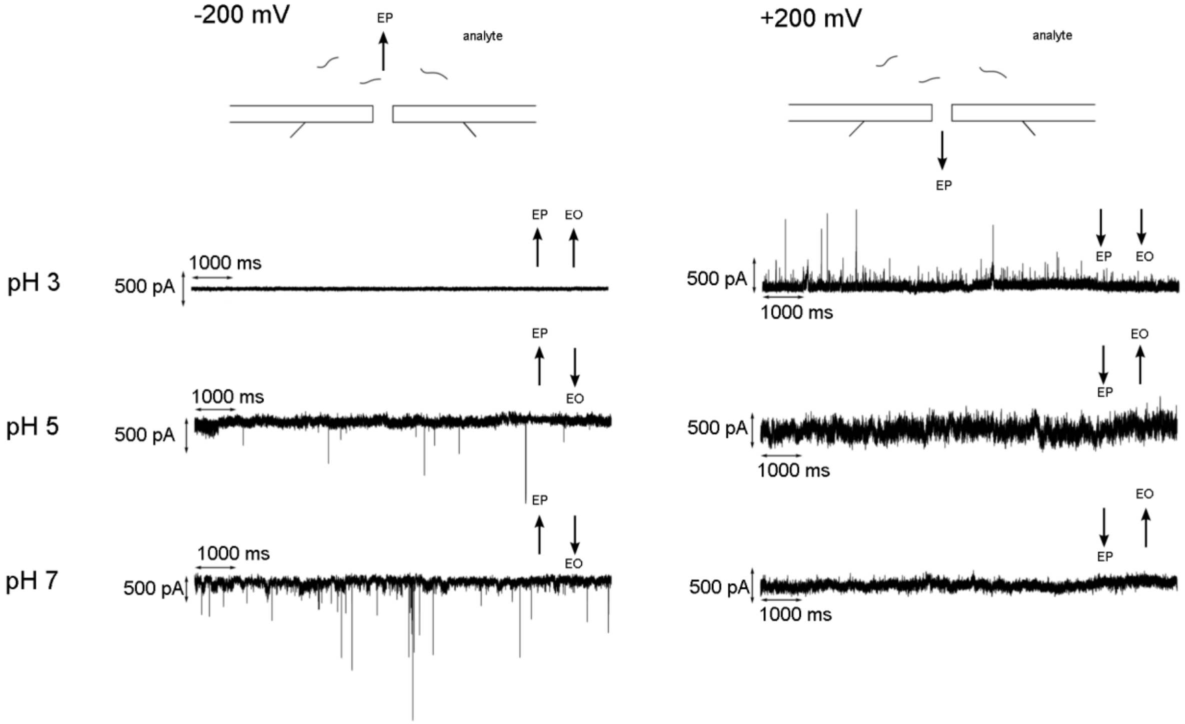

Supplementary Figure 7. Nanopore alginate events as a function of solution pH. Representative current events of **A1** alginate at pH 3,5 and 7 at positive and negative 200 mV voltage differences applied for 1-hour each in the same ~8 nm diameter pore at 1M KCl. The schematic shows the placement of the (anionic) analyte relative to the nanopore and direction of electrophoresis (EP), and the direction for electroosmosis (EO) is given next to the current traces.

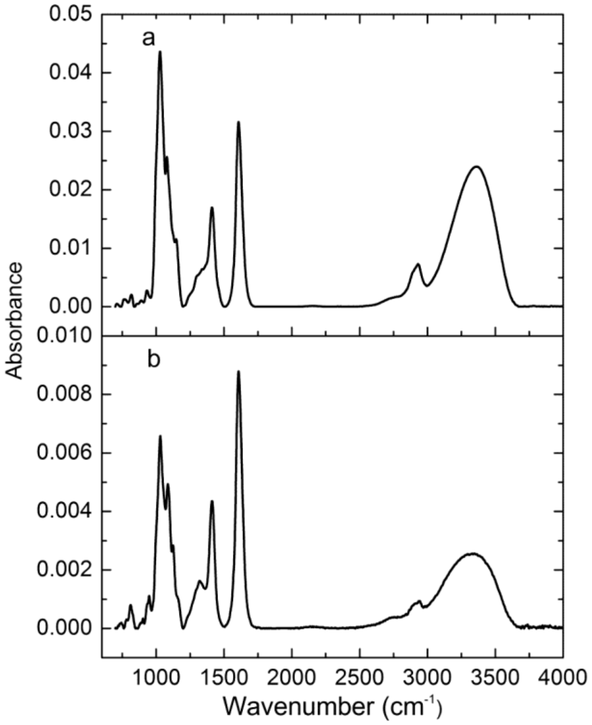

Supplementary Figure 8. Infrared spectra of alginate samples. The intensity of the peaks near 1400 and 1600 cm^{-1}, relative to the remainder of the spectrum, are consistent with a lesser proportion of carboxylic acid salt residues in (a) **A1** than in (b) **A2**. Comparison of the intensity of the guluronic (G) unit absorption at ~1025 cm^{-1} to the mannuronic (M) unit absorption at ~1100 cm^{-1} allows calculation of the M/G ratio that varies with particular alginate source.[20] Using this approach, alginate **A1** was determined to be ~63%G/37%M, and alginate **A2** was ~57%G/43%M. These relative proportions were supported by additional analysis: in Supplementary Figure 3b, the particular alginate lyase was a mannuronic lyase, so that the greater absorption from the digestion of **A2** than **A1** was consistent with a greater proportion of *M* in **A2**.

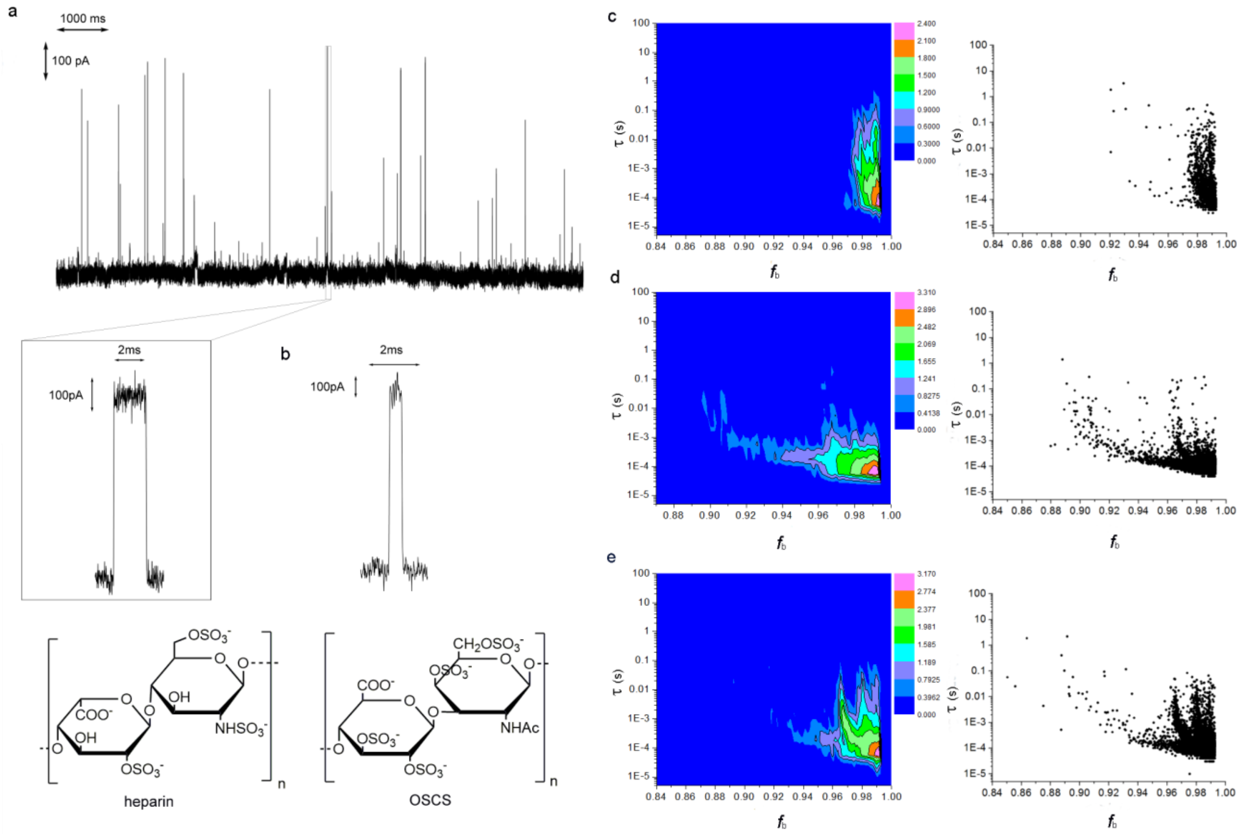

Supplementary Figure 9. Heparin and OSCS events. A representative (a) segment of a heparin induced-current trace using a ~9.4 nm-diameter pore with a magnified current event from the same trace, and from (b) OSCS through the same pore in response to a +200 mV applied voltage in 4 M KCl at pH ~7. (c) Contour (left) and scatter (right) plots for heparin (top), OSCS (middle) and heparin contaminated with OSCS (bottom) through a ~13 nm diameter pore.

Recognition Flag Generation

Recognition flag generation was done using custom codes written in Mathematica 11.0.1.0 (Wolfram, Champaign, IL). (1) All individual events were histogrammed with respect to f_b using a bin width of 0.0025 (using nanopores with diameters from ~8-13 nm, and determined using the USP heparin data). (2) Any bin with counts below 0.5% of the maximum bin count were removed, and all counts were then normalized. (3) The OSCS identification threshold was taken to be at the nearest bin at the distance of three standard deviations (after the 0.5% filter) from the bin with the maximum number of counts. (4) When events had been detected at f_b below this threshold, the recognition flag was set to red to signal the presence of OSCS; it was otherwise left white.

(5) All individual events were then histogrammed with respect to the logarithm ($\log_{10}$) of the event duration (τ) using a bin width of 0.25 (here, determined using the USP OSCS data). (6) The same 0.5% filter was applied to these histograms, which then had their counts normalized. (7) The event duration threshold was taken to be the nearest bin at the distance of three standard deviations (after the 0.5% filter) from the bin with the maximum number of counts. (8) When events had been detected at $\log_{10} \tau$ above this threshold, the recognition flag was set to red to signal the presence of heparin; it was otherwise left white.

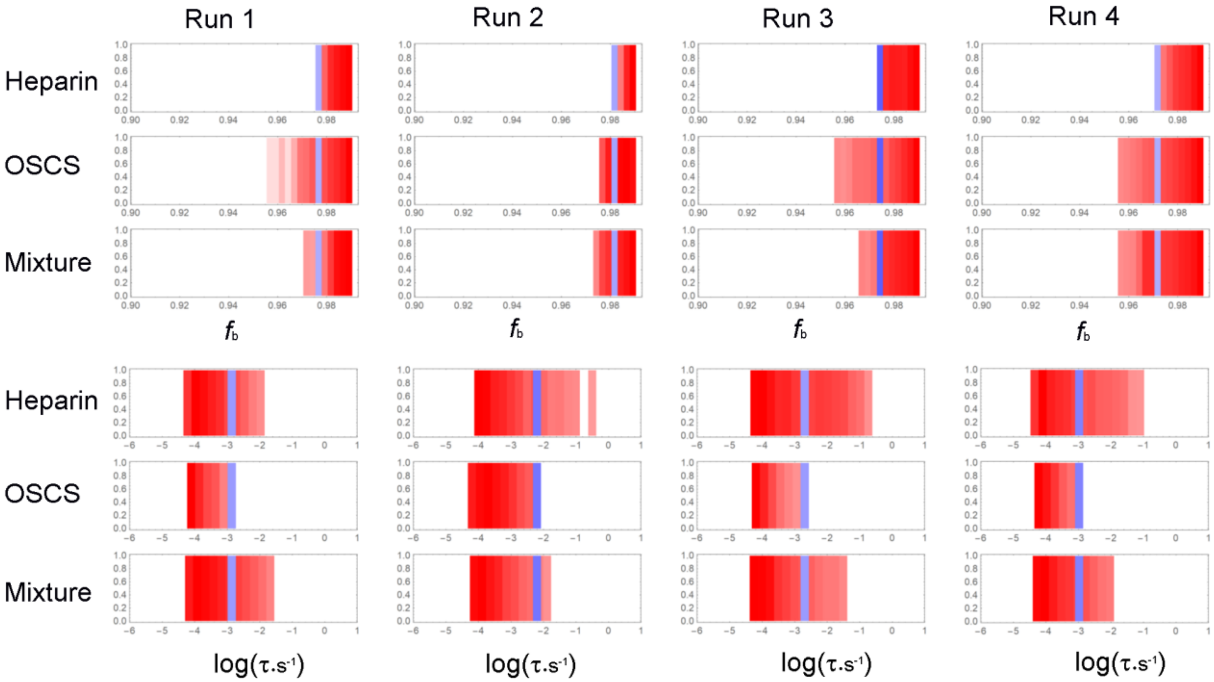

Supplementary Figure 10. Heparin and OSCS recognition flag statistics. Hue plots show the outcomes of recognition flag generation (and measurement statistics—see procedure detailed above) after steps 3 (top) and 7 (bottom), based on $f_b = \langle i_b \rangle / \langle i_0 \rangle$ and $\log_{10} \tau$ of the individual events. The identification threshold, determined by the measurement statistics of each run, is given by the blue line. The corresponding final recognition flags, showing successful detection of the toxic OSCS impurity across four independent trials in ~8, 9, 9, and 13 nm (left to right), are shown in Figure 5. See also Supplementary Figure 4 e,f.

Supplementary References

1. Viskov C, *et al.* Isolation and Characterization of Contaminants in Recalled Unfractionated Heparin and Low-Molecular-Weight Heparin. *Clin Appl Thromb-Hem* **15**, 395-401 (2009).

2. Trivedi DM. FABRICATION AND CHARACTERIZATION OF SILICON NITRIDE NANOPORES. M.Sc. Thesis. The University of British Columbia (2009).

3. Kwok H, Briggs K, Tabard-Cossa V. Nanopore Fabrication by Controlled Dielectric Breakdown. *PLoS ONE* **9**, e92880 (2014).

4. Frament CM, Dwyer JR. Conductance-Based Determination of Solid-State Nanopore Size and Shape: An Exploration of Performance Limits. *J Phys Chem C* **116**, 23315-23321 (2012).

5. Kowalczyk SW, Grosberg AY, Rabin Y, Dekker C. Modeling the conductance and DNA blockade of solid-state nanopores. *Nanotechnology* **22**, 315101 (2011).

6. Lee C, Joly L, Siria A, Biance A-L, Fulcrand R, Bocquet L. Large Apparent Electric Size of Solid-State Nanopores Due to Spatially Extended Surface Conduction. *Nano Lett* **12**, 4037-4044 (2012).

7. Huggins ML. The Viscosity of Dilute Solutions of Long-Chain Molecules. IV. Dependence on Concentration. *J Am Chem Soc* **64**, 2716-2718 (1942).

8. Clementi F, Mancini M, Moresi M. Rheology of alginate from Azotobacter vinelandii in aqueous dispersions. *J Food Eng* **36**, 51-62 (1998).

9. Armstrong JK, Wenby RB, Meiselman HJ, Fisher TC. The Hydrodynamic Radii of Macromolecules and Their Effect on Red Blood Cell Aggregation. *Biophys J* **87**, 4259-4270 (2004).

10. Foulger JH. THE USE OF THE MOLISCH (α-NAPHTHOL) REACTIONS IN THE STUDY OF SUGARS IN BIOLOGICAL FLUIDS. *J Biol Chem* **92**, 345-353 (1931).

11. Hallal JLJ, Lucho AMS, Gonçalves RS. Electrochemical polymerization of furfural on a platinum electrode in aqueous solutions of potassium biphthalate. *Mat Res* **8**, 23-29 (2005).

12. Skidmore MA, Guimond SE, Dumax-Vorzet AF, Yates EA, Turnbull JE. Disaccharide compositional analysis of heparan sulfate and heparin polysaccharides using UV or high-sensitivity fluorescence (BODIPY) detection. *Nat Protocols* **5**, 1983-1992 (2010).

13. Draréni J, Roy S. A Simple Oriented Mean-Shift Algorithm for Tracking. In: *Image Analysis and Recognition* (ed^(eds Kamel M, Campilho A). 1 edn. Springer-Verlag Berlin Heidelberg (2007).

14. Smeets RMM, Kowalczyk SW, Hall AR, Dekker NH, Dekker C. Translocation of RecA-Coated Double-Stranded DNA through Solid-State Nanopores. *Nano Lett* **9**, 3089-3095 (2009).

15. Meller A, Nivon L, Branton D. Voltage-driven DNA translocations through a nanopore. *Phys Rev Lett* **86**, 3435-3438 (2001).

16. Meller A, Nivon L, Brandin E, Golovchenko J, Branton D. Rapid nanopore discrimination between single polynucleotide molecules. *P Natl Acad Sci USA* **97**, 1079-1084 (2000).

17. Krasilnikov OV, Rodrigues CG, Bezrukov SM. Single Polymer Molecules in a Protein Nanopore in the Limit of a Strong Polymer-Pore Attraction. *Phys Rev Lett* **97**, 018301 (2006).

18. Steinbüchel A, Rhee SK. *Polysaccharides and Polyamides in the Food Industry*. Wiley (2005).

19. Sellimi S, *et al.* Structural, physicochemical and antioxidant properties of sodium alginate isolated from a Tunisian brown seaweed. *Int J Biol Macromol* **72**, 1358-1367 (2015).

20. Pereira L, Sousa A, Coelho H, Amado AM, Ribeiro-Claro PJA. Use of FTIR, FT-Raman and 13C-NMR spectroscopy for identification of some seaweed phycocolloids. *Biomol Eng* **20**, 223-228 (2003).
